# TREM2 activation on microglia promotes myelin debris clearance and remyelination in a model of multiple sclerosis

**DOI:** 10.1007/s00401-020-02193-z

**Published:** 2020-08-09

**Authors:** Francesca Cignarella, Fabia Filipello, Bryan Bollman, Claudia Cantoni, Alberto Locca, Robert Mikesell, Melissa Manis, Adiljan Ibrahim, Li Deng, Bruno A. Benitez, Carlos Cruchaga, Danilo Licastro, Kathie Mihindukulasuriya, Oscar Harari, Michael Buckland, David M. Holtzman, Arnon Rosenthal, Tina Schwabe, Ilaria Tassi, Laura Piccio

**Affiliations:** 1 Department of Neurology, Washington University School of Medicine, 660 S. Euclid Avenue, Campus Box 8111, St. Louis, MO 63110 USA; 2 Department of Biomedical Sciences, Humanitas University, Via Rita Levi Montalcini 4, Pieve Emanuele – Milan, 20090 Italy; 3 Alector, 131 Oyster Point Blvd #600, South San Francisco, CA 94080 USA; 4 Department of Anesthesiology, First Affiliated Hospital of Soochow University, Suzhou, 215006 Jiangsu China; 5 Department of Psychiatry, Washington University School of Medicine, St. Louis, MO 63110 USA; 6 Hope Center for Neurological Disorders, Washington University School of Medicine, St. Louis, MO 63110 USA; 7 NeuroGenomics and Informatics, Washington University School of Medicine, St. Louis, MO 63110 USA; 8 ARGO Open Lab Platform for Genome sequencing, AREA Science Park, Padriciano 99, 34149 Trieste, Italy; 9 Brain and Mind Centre, University of Sydney, 94 Mallett St Camperdown, Sydney, NSW 2050 Australia; 10 Present Address: Alector, 131 Oyster Point Blvd #600, South San Francisco, CA 94080 USA

## Abstract

Multiple sclerosis (MS) is an inflammatory, demyelinating, and neurodegenerative disease of the central nervous system (CNS) triggered by autoimmune mechanisms. Microglia are critical for the clearance of myelin debris in areas of demyelination, a key step to allow remyelination. TREM2 is expressed by microglia and promotes microglial survival, proliferation, and phagocytic activity. Herein we demonstrate that TREM2 was highly expressed on myelin-laden phagocytes in active demyelinating lesions in the CNS of subjects with MS. In gene expression studies, macrophages from subjects with TREM2 genetic deficiency displayed a defect in phagocytic pathways. Treatment with a new TREM2 agonistic antibody promoted the clearance of myelin debris in the cuprizone model of CNS demyelination. Effects included enhancement of myelin uptake and degradation, resulting in accelerated myelin debris removal by microglia. Most importantly, antibody-dependent TREM2 activation on microglia increased density of oligodendrocyte precursors in areas of demyelination, as well as the formation of mature oligodendrocytes thus enhancing remyelination and axonal integrity. These results are relevant as they propose TREM2 on microglia as a potential new target to promote remyelination.

**Electronic supplementary material:**

The online version of this article (10.1007/s00401-020-02193-z) contains supplementary material, which is available to authorized users.

## Introduction

Multiple sclerosis (MS) is a chronic demyelinating disease of the central nervous system (CNS), whose pathogenesis involves inflammatory and neurodegenerative processes. Current MS immunomodulatory treatments target CNS inflammation, while therapies capable of regenerating myelin and halting disease progression are lacking [28]. Oligodendrocyte precursor cells (OPCs) are multipotent progenitor cells widely distributed in the CNS that could differentiate into mature oligodendrocytes (OLs) to sustain remyelination. In MS, impaired generation of OLs from OPCs leads to persistent demyelination, myelin debris accumulation, and axonal damage which clinically manifests as neurological disability [11]. Efficient myelin debris removal and clearance by phagocytic cells are critical to eliminate inhibitory signals interfering with OPC activation, recruitment to the site of demyelination and/or differentiation into myelinating mature OLs [14, 26, 27].

Microglial cells and infiltrating monocytes/macrophages can have a dual role in MS lesions. They could contribute to myelin damage and lesion expansion, or they may have a protective role by clearing myelin debris, reducing inflammation and secreting regenerative factors promoting remyelination [48]. A critical modulator of microglia functions is the triggering receptor expressed on myeloid cells-2 (TREM2), an innate immune receptor expressed by several myeloid cells including brain microglia [23, 45]. TREM2 is a phospholipid sensing receptor known to sustain microglial cell activation and expansion in response to demyelination or amyloid plaques in Alzheimer’s disease (AD) [8, 42, 50]. Recently, TREM2 was proposed to be a key transcriptional regulator of cholesterol metabolism during chronic phagocytic activity for myelin clearance in response to demyelination [35].

TREM2 binds on the membrane to the DNAX-activation protein 12 (DAP12) which is an adaptor protein required for TREM2 surface expression and intracellular signaling [47]. TREM2 engagement leads to DAP12 phosphorylation, followed by recruitment and activation of the spleen-associated tyrosine kinase (SYK), resulting in downstream signaling events leading to proliferation, survival, phagocytosis, and secretion of cytokines and chemokines [47]. Homozygous loss-of-function mutations in TREM2 or DAP12 genes cause Nasu–Hakola disease (NHD), a rare genetic disorder characterized by fatal presenile dementia and bone cysts [38, 39]. Neuropathological findings in NHD include loss of myelin and axons in the brain, with reactive astrocytosis and microglial activation [25]. Heterozygous TREM2 gene variants were reported to increase the risk for AD and other neurodegenerative diseases (frontotemporal dementia, Parkinson’s disease and amyotrophic lateral sclerosis) [7, 16, 20, 43].

Here, we explored the effect of antibody-mediated TREM2 activation on microglia in a well-established toxin-induced model of demyelination in the CNS resulting from exposure to the copper chelator cuprizone (CPZ) in the diet [31]. In this model, oligodendrocyte degeneration in the brain is followed by a robust microglial response consisting of activation, proliferation and clearance of myelin debris [17]. These events lead to OPC recruitment, differentiation into mature OLs, and then remyelination which is almost complete within few weeks from toxin withdrawal [31]. We show that treatment with a TREM2-agonistic antibody was able to enhance myelin debris clearance by microglia in vivo in the CPZ-model and by bone marrow-derived macrophages (BMDM) in vitro. Most importantly, these events resulted in increased OPC recruitment and differentiation into mature OLs eventually accelerating remyelination in vivo and preserving axonal health. This provides the proof of concept that antibody-mediated TREM2 activation could promote remyelination, suggesting this as a potential novel therapeutic avenue in MS and other demyelinating disorders.

## Materials and methods

### Mice

*Trem2*^+*/*+^, *Trem2*^+*/*^,^−^*and Trem2*^−*/*−^ mice (backcrossed 12 generations to the C57BL/6 background) were obtained from Professor Marco Colonna (Washington University in St. Louis). These three strains were bred in parallel. Animals were housed in accordance with University and National Institutes of Health (NIH) guidelines, and animal protocols were approved by the Washington University Animal Studies Committee (study approval number: 20180056). Mice were maintained under controlled conditions (19–22 °C and a in a 12-h light/dark cycle with unrestricted access to food and water). Experiments performed by Alector (Fig. 2 and Supplementary Fig. 1a and c) used the *Trem2*^−*/*−^ mouse colony originally generated by the trans-NIH KnockOut Mouse Project (KOMP). Frozen sperms were obtained from the UC Davis KOMP repository, and a colony of mice was established at UC Davis.

### Antibody generation

Monoclonal antibodies targeting mouse TREM2 were generated by immunizing mice genetically deficient of TREM2 (*Trem2*^−*/*−^) with recombinant TREM2 protein and hybridoma generation, as well as by a yeast display campaign (performed by Adimab). AL002a was screened for TREM2 specificity by selecting for binding to wild-type (*Trem2*^+*/*+^), but not *Trem2*^−*/−*^ BMDM.

### Mouse model of CPZ‑induced demyelination

Six- to eight-week-old *Trem2*^+*/*+^, *Trem2*^+*/−*^, and *Trem2*^−*/*−^ mice were fed a 0.2% Bis-(cyclohexanone) oxaldihydrazone (cuprizone) diet (5C5N: Modified PicoLab^®^ Rodent w/0.2% Cuprizone, TestDiet. Cuprizone from Alpha Aesar, A10628) for 4 weeks (WK 4) or for 4 weeks followed by 3 days (WK 4 + 3D), 7 days (WK 4 + 7D), or 14 days (WK 4 + 14D) on regular chow (PicoLab Rodent Diet 20 #5053, Purina). For the full duration of the experiment mice were injected intraperitoneally (i.p.) once a week with the anti-TREM2 antibody or the control antibody at a dose of 80 mg/kg. The first injection with the antibodies was performed 4 days before beginning the CPZ-diet.

### Quantification of antibody levels in the brain

Six- to eight-week-old *Trem2*^+*/−*^ mice were injected i.p. with AL002a. 48 h post injection, brains were removed after perfusion with PBS, micro-dissected to isolate the corpus callosum (CC) and the cortex (Ctx), and immediately frozen in liquid nitrogen. Tissues were solubilized in N-Per lysis buffer (87792, ThermoFisher) and cell protein content was measured using the Pierce bicinchoninic acid (BCA) protein assay kit (23227, Thermo Scientific). A mouse Trem-2b/Fc Chimera (R&D Systems) was used as a capture antibody and coated overnight at 4 °C on 96-well Meso Scale Discovery (MSD) plates in PBS. After washing, wells were blocked for 1 h at 37 °C with binding buffer (3% BSA in PBS). Titration of samples and standards were incubated for 1 h at room temperature (RT) on a shaker at 500 rpm. For detection (0.5 mg/ml) of Sulfo-TAG goat anti-mouse IgG (MSD) were added to the plate. After washing, read buffer was added and the plate was read on the Sector Imager. Washes between the different steps were done three times with 0.05% Tween 20 in PBS. Antibody content was normalized to serum and protein content.

### Bone marrow-derived macrophages

Bone marrow-derived macrophages (BMDM) were obtained by flushing tibial and femoral marrow cells with cold PBS 2% FBS. Red blood cells were lysed using ACK lysing buffer (Thermo Fisher), and after two washes in PBS 2% FBS, the cells were re-suspended in complete media (RPMI, 10% FBS, Pen/Strep, l-glutamine, non-essential amino acid) with (50 ng/ml) murine M-CSF (m-M-CSF) to obtain differentiated macrophages after 6 days. Adherent macrophages were detached with 1 mM EDTA in PBS.

### Immunoprecipitation

Immunoprecipitation in vitro: before stimulation, BMDM were starved for 4 h in RPMI with 1% FBS. 10 × 10^6^ cells were incubated for 15 min at 4 °C with AL002a or control antibody (1 µg for 10^6^ cells). Cells were then washed and incubated at 37 °C in the presence of goat-anti mouse IgG (1.5 µg for 1 × 10^6^ cells). After stimulation, cells were lysed with lysis buffer (1% *n*-dodecyl-β-d-Maltoside, 50 Mm Tris–HCl (pH 8.0), 150 mM NaCl, 1 mM EDTA, 1.5 mM MgCl2, 10% glycerol, protease, and phosphatase inhibitors) and immunoprecipitated with an anti-TREM2 antibody that binds a different domain (rat anti-h/m-TREM2, clone 237920, R&D system). Immunoprecipitation in vivo: 6- to 8-week-old C57BL/6 mice were injected i.p. with 3 ml of 3% thioglycollate. After 3 days, when the peritoneal cavity was enriched with CD11b^+^F4/80^+^ macrophages expressing TREM2, mice were injected with control or TREM2-specific antibodies (40 mg/kg). 24 h after antibody injection, peritoneal macrophages were collected, immediately lysed in the lysis buffer previously described, and immunoprecipitated with rat anti-h/m TREM2 antibody described above (R&D System, clone 237920). Then, for both in vitro and in vivo experiments, precipitated proteins were fractionated by SDS-PAGE in non-reducing conditions, transferred to PVDF membranes, and probed with an anti-phosphotyrosine antibody (4G10, Millipore). TREM2 is not detected in non-reducing condition. To confirm that all substrates were adequately immunoprecipitated, whole cell lysates from each sample were also fractionated by SDS-PAGE in reducing condition and immunoblotted with an anti-actin antibody (actin, sc-47778 Santa Cruz).

### Myelin production

Human myelin was prepared as previously described [34] and stored in lyophilized form at − 80 °C. Prior to use, myelin was suspended in DMEM to a final concentration of (2 mg/ml) and dissolved by vortexing and sonicating. Myelin was then irradiated with 10,000 RADS to achieve sterility. Aliquots were stored at − 80 °C for further use.

### NFAT-Luciferase reporter assay

A stable BW5147.G.1.4 (ATCC^®^ TIB48™) (BWZ) cell line expressing both mouse TREM2 and DAP12 was generously provided by the Seaman lab [10]. This line was infected with a Cignal PLenti NFAT-Luciferase virus (Qiagen) to generate a stable mouse TREM2 reporter cell line, able to induce luciferase signaling upon TREM2 activation. The activity of the reporter was validated using PMA (0.05 μg/ml) and ionomycin (0.25 μM). To test whether TREM2 antibody induced signaling, 5 µg/ml of soluble AL002a, or control antibody was added to each well of 96-well culture plates together with 100,000 cell/well and incubated for 4–6 h at 37 °C in Dulbecco’s Modified Eagle Medium (DMEM). Luciferase activity was measured by removing media and adding 50 μl of PBS and 50 μl of OneGlo Reagent (Promega) to each well and incubating for 3 min at room temperature on a plate shaker to lyse the cells. Luciferase signal was measured using a BioTek plate reader. Data were analyzed using GraphPad Prism.

To test myelin-induced signaling, human myelin was diluted in PBS to 200 µg/ml, titrated onto a 96-well tissue culture plate, and incubated overnight at 4 °C. The next morning, the solution was removed, and plates were washed three times with 200 µl PBS. Plates were air dried and BWZ cells (with or without antibodies) were added, incubated, and analyzed as described above.

### Myelin phagocytosis and degradation assays

BMDM obtained from *Trem2*^+*/−*^ mice were seeded in 8-well chamber slides (35,000 cells/well) (154534 Nunc, Lab-Tek) in normal medium as described before. After 1 day, media was substituted with reduced FBS media (5% FBS) and macrophages were pre-incubated with AL002a or control antibody (10 µg/ml) 3 h before starting the experiments. For phagocytosis assays, BMDM were incubated with human myelin (20 µg/ml) for 30 min, 1 h, or 3 h, and after incubation, cells were washed in PBS and fixed in 4% PFA. For degradation assays, BMDM were incubated with human myelin (20 µg/ml) for 2 h, then washed thoroughly with PBS, and again put in culture in reduced FBS media. Cells were left in culture for 1 h, 24 h, or 48 h, then washed in PBS, and fixed in 4% PFA. For intracellular staining, cells were permeabilized and blocked for 60 min at RT in 5% horse serum and 0.1% saponin in PBS, and incubated at 4 °C overnight with primary antibodies Rt anti-MBP (Abcam, ab7349, 1:100) and Gt anti-Iba1 (Novus, NB100-1028, 1:250) diluted in PBS and 5% horse serum.

### Mouse tissue processing and histological analyses

Mice were perfused with 4% paraformaldehyde. Mouse brains were removed and post-fixed in 4% PFA for 24 h, followed by immersion in 30% sucrose for 48 h, then embedded in Optimal Cutting Temperature (OCT). 5-μm sections were placed on glass slides and stained with solochrome cyanine to confirm the presence of a lesion as previously described [22]. Sections were stained with the following primary antibodies: Rb anti-dMBP (Millipore, ab5864, 1:2000), Rb anti-Iba1 (Wako, 019-19741, 1:600), Gt anti-Iba1 (Novus, NB100-1028, 1:250), Rt anti-LAMP1 (Abcam, ab25245, 1:500), Rt anti-CD68 (Invitrogen, 14-0681-82, 1:300), Rb anti-PDGFRα (ThermoFisher, PA5-16742, 1:50), Rb anti-OLIG2 (Milipore, AB9610, 1:300), Ms anti-CNPase (Abcam, ab6319, :100), Shp anti-BrdU (Abcam, ab1893, 1:250), Rt anti-GFAP (ThermoFisher, 13-0300, 1:200), and Ms anti-SMI-31 (Biolegend, 801603, 1:1000). AlexaFluor-conjugated secondary antibodies (Invitrogen, 1:1000) were used. Some of the images were acquired with a Nikon Eclipse 90*i* fluorescent and bright field microscope equipped with 10 × and 20 × zoom objectives and analyzed with Metamorph 7.7 software. CNPase, dMBP, and GFAP were analyzed as the percentage area of positive staining (number of positive pixels/mm^2^) within the region of interest. Iba1, PDGFRα, BrdU, and OLIG2 were quantified as the density of cells in the region of interest (number of cells/mm^2^). LAMP1 and CD68 were analyzed as the percentage area of LAMP1^+^Iba1^+^ and CD68^+^Iba1^+^ staining (number of positive pixels/mm^2^) and then normalized on the percentage of Iba1^+^ staining (number of positive pixels/mm^2^) within the region of interest. For confocal analysis, images were acquired with an Olympus FV1200 laser scanning confocal microscope (Olympus-America Inc., Waltham, MA) equipped with a PlanApoN 60 ×, 1.4 NA super corrected oil objective. The Olympus FV1200 confocal microscope was equipped with five detectors: two spectral and one filter-based and two gallium arsenide phosphide (GaAsP) photo-multiplier tubes (PMTs). The 405-, 488-, and 559-nm diode lasers and 635-nm HeNe (helium neon) lasers were used with an optimal pinhole of 1 airy unit to acquire images. Images were finally processed with ImageJ and Imaris Software (Bitplane, Switzerland).

### Transmission electron microscopy and g-ratio quantification

Mice were perfused with PBS, brains were removed, and immersion fixed in 2% PFA, 2.5% Glutaraldehyde, and 0.1 M PBS. 50 μm sagittal brain sections were cut using a vibratome, then fixed in osmium tetroxide in 0.1 M PBS (EMS, 19100), followed by dehydration in ethanol and infiltration of Spurr’s resin. Tissues were embedded using Spurr’s resin and aclar film. After polymerizing, the corpus callosum was dissected from the tissue and attached to a pre-made Spurr’s resin block, then sectioned using a DiATOME ultra 45° diamond knife and a LEICA Ultracut UC7. 90-nm sections were cut and picked up onto 200 hex mesh, formvar-carbon coated copper grids (Ted Pella, 01800-F), and stained with uranyl acetate and lead citrate. Images were captured using a JEOL 1200 EX II Transmission Electron Microscope with AMT digital camera. Remyelination was analyzed by counting the number of naked axons and the number of myelinated axons per field, with a minimum of ten fields being analyzed. The g-ratio was quantified by dividing the axonal diameter by the myelinated fiber diameter. Thirty myelinated axons were randomly analyzed across multiple fields per mouse to calculate the g-ratio.

### In vivo myelin engulfment quantification

Fixed brain slices were permeabilized for 45 min at RT in PBS 0.1% Triton X-100, followed by 1 h RT in blocking solution (2% BSA 0.1% Triton X-100 in PBS) and overnight incubation with primary antibody for Rb anti-dMBP (Millipore, ab5864, 1:2000), Gt anti-Iba1 (Novus, NB100-1028, 1:250) and Rt anti-CD68 (Invitrogen, 14-0681-82, 1:300) at 4 °C. Upon washing, sections were incubated 2 h at RT with Alexafluor-conjugated secondary antibodies (Invitrogen, 1:1000). Images were acquired with an Olympus FV1200 laser scanning confocal microscope (Olympus-America Inc., Waltham, MA) with 2 × digital zoom, and a z-step size of 0.33 µm. Z-stacks ranged from 4 to 5 µm in thickness. Images were processed and analyzed by Imaris Software (Bitplane, Switzerland). CD68 and Iba1 volume was quantified by applying 3D surface rendering of confocal z-stacks in their respective channels, using identical settings (fix thresholds of intensity and voxel) within each experiment. Each confocal acquisition contained an equal number of images from the CC of mice treated with AL002a and control antibody. For quantification of dMBP engulfment by microglia, only dMBP signals present within microglial CD68^+^ structures were considered. To this end, a new channel for ‘‘engulfed dMBP’’ was created, by using the mask function in Imaris, masking the dMBP signal within CD68^+^ structures. Quantification of volumes for ‘engulfed dMBP in CD68’ was performed following the ‘3D Surface rendering of engulfed material’ protocol previously published [44]. To account for variations in cell size, the amount of ‘engulfed dMBP in CD68’ was normalized to the total volume of the phagocyte in each field (given by Iba1^+^ total volume). Total dMBP volume per field from the same confocal z-stacks was also quantified following the same protocol.

### Neurofilament light detection

Mouse blood was collected into EDTA tubes (Sarstedt 201341102) with a capillary tube (Sarstedt 201278100), spun at 15,000×*g* for 7 min at 4 °C, and the top plasma layer was transferred to a 1.5 ml tube and stored at − 80 °C. Frozen plasma samples were thawed at room temperature, diluted tenfold, and run on a SIMOA HD-X (Quanterix) using the Simoa NF-light advantage kit (Quanterix 103186) according to the manufacturer’s protocol.

### BrdU used as a marker of proliferation

Microglia and OPC proliferation in vivo was measured by 5-bromo-2′-deoxyuridine (BrdU) incorporation (Sigma, B5002). BrdU was administered (25 mg/kg) by intra peritoneal injection every 12 h starting 4 days before collecting the brains.

### Microglia isolation and flow cytometry

Microglia isolation was carried out following a published protocol [3] with modifications. The whole procedure was done on ice with cold buffers and centrifuges at 4 °C. Briefly, anesthetized mice were perfused intracardially with ice-cold Hank’s balanced salt solution (HBSS), and the CC and hippocampi were dissected under a stereotactic microscope. The different areas were mechanically homogenized and digested in ice-cold Accutase (Millipore) on a wheel for 20 min at 4 °C. After spinning the tubes for 1 min at 2000*g*, pellets were resuspended in cold Hibernate buffer (Thermo Fisher). The cell suspension was then transferred to pre-chilled 15 ml tubes and passed through a pre-wet (with Hibernate buffer) 70 µm cell strainer (PluriStrainer Mini). Cell suspensions were then spun down at 300*g* for 10 min and pellets were resuspended in PBS + 2% FBS, counted, and labeled with a combination of the following conjugated antibodies: CD11b-PeCy7 (clone M1/70), CD45-Alexa 700 (clone 30-F11), P2ry12-PE (clone S16007D), CD80-FITC (clone 16-10A1), CD86-BV421 (clone GL-1), and zombie acqua (Biolegend). Dead cells were excluded by selecting the zombie aqua negative cells. FACS analysis of the microglial profile was performed by gating CD11b^+^CD45^int^ cells. Appropriate IgG isotype control antibodies were used for all staining. FACS analysis was performed on a FACS Fortessa machine (BD Biosciences), and data were analyzed with FlowJo Software (TreeStar).

### Human tissue and analysis

Twenty fresh-frozen blocks of post-mortem CNS tissue from eight MS patients and four controls with non-neurological diseases were obtained from The Neuroinflammatory Disease Tissue Repository at Washington University St. Louis. Demographic and clinical  characteristics of the donors of human brain tissues at the time of collection are indicated in Table 1. 5 μm sections were stained with Solochrome Cyanine and Oil Red O to look at myelin and lipid-laden macrophages, respectively. Active lesions were characterized as tissue areas with marked demyelination and the presence of lipid-laden macrophages. Tissues were then stained with Gt anti-human TREM2 (R&D Systems, AF1828, 1:200) and Rb anti-Iba1 antibody (Wako, 019-19741, 1:600). AlexaFluor-conjugated secondary antibodies were used (Invitrogen, 1:1000). Histological images were acquired using the Nanozoomer microscope at the Hope Center for Neurological Disorders at Washington University. For confocal analysis, images were acquired with a Zeiss LSM880 Airyscan laser scanning confocal microscope (Carl Zeiss Inc., Thornwood, NY) equipped with 63X, 1.4 numerical aperture (NA) Zeiss Plan Apochromat oil objective. The system is equipped with a unique scan head, incorporating a high-resolution galvo scanner along with two PMTs and a 32-element spectral detector. ZEN 2.3 black edition software was used to obtain Z-stacks through the entire height of the cells with confocal Z-slices of 5 µm (63 ×) and an interval of 0.347 µm. Images taken were optimized for 1 airy unit using the 405-nm diode, 488 nm Argon, and 561 nm diode and 633 m HeNe (helium neon) lasers. In addition, the Airyscan unit provides sub-diffraction limited imaging down to 120 nm resolution. Quantitative PCR analysis was performed on adjacent tissue sections.

**Table 1:** Human CNS tissues from autopsied multiple sclerosis and healthy control subjects

Patient ID	Age/gender	Disease duration at death	Post mortem interval	Cause of death	MS diagnosis	Tissue	Lesion type
38	39/F	NA	4	CNS malignant lymphoma	NA	Cerebellum	Control
10	41/M	NA	24	Heart failure	NA	Spinal cord Spinal cord Cerebrum	Control Control Control
52	69/F	NA	43	Sepsis	NA	Cerebrum	Control
32	56/F	NA	18	Acute myocardial infarction	NA	Spinal cord Cerebrum	Control Control
46	77/F	Unknown	16	Not reported	SPMS	Spinal cord	MS NAWM
45	66/F	32	29	Not reported	SPMS	Brainstem Cerebrum	MS NAWM MS NAWM
77	41/F	15	12	Complication from Type 1 diabetes mellitus	RRMS	Brainstem Spinal cord Cerebrum	MS NAWM MS NAWM MS NAWM
40	60/M	14	9	Respiratory failure	SPMS	Spinal cord	MS active
30	54/F	17	7	Pneumonia	SPMS	Spinal cord	MS active
71	50/F	13	20	Not reported	SPMS	Spinal cord Spinal cord	MS active MS active
21	69/F	29	6	Metastatic colon cancer	PPMS	Spinal cord Spinal cord	MS active MS active
59	54/F	22	8	Pneumonia	SPMS	Spinal cord	MS active

*NA* not applicable, *SPMS* secondary progressive multiple sclerosis, *RRMS* relapsing remitting multiple sclerosis, *PPMS* primary progressive multiple sclerosis, *NAWM* normal appearing white matter

### Quantitative PCR

RNA was extracted using the RNeasy Micro Kit (Qiagen), converted into cDNA using the High-Capacity cDNA Reverse Transcription Kit (Applied Biosystems), and used at 20–40 ng in quantitative real-time PCR (qPCR) analysis. The DDCt method was applied to determine differences in gene expression levels after normalization to the arithmetic mean of *GAPDH* as internal standards. The TaqMan probes used are the following: for human studies: *GAPDH* (Hh_99999905_m1) and *TREM2* (Hs00219132_m1); for mouse studies: *Gapdh* (Mm99999915_g1); *Olig2* (Mm01210556_m1); *Mbp* (Mm01266402_m1); *Cnp* (Mm01306641_m1); *Mog* (Mm00447824_m1); and *Plp* (Mm01297210_m1). All data are the mean of duplicates, and the standard errors of the mean was calculated between duplicates or triplicates. Real-time PCR was performed using an ABI 7000 Real-Time PCR System (Applied Biosystems).

### Human macrophage cultures from Nasu–Hakola disease (NHD) patients

Peripheral blood mononuclear cells (PBMCs) were purified from peripheral blood samples on Ficoll-Paque PLUS density gradient (Amersham Biosciences, Piscataway, NJ). Samples were from three NHD patients and three control subjects, their characteristics at the time of collection are indicated in Table 2. To generate macrophages, PBMCs were seeded in RPMI without FBS for 2 h, then PBMCs were thoroughly washed with PBS, and cultured in complete media (RPMI, 10% FBS, Pen/Strep, l-glutamine, non-essential amino acid) supplemented with (50 ng/ml) of recombinant human M-CSF (300-25, Peprotech) for 7 days at 37 °C in 5% CO_2_. Cells were then harvested and immediately lysed in RLT Buffer; RNA was extracted using the RNeasy Micro Kit (Qiagen).

**Table 2:** Genetic and demographic characteristics of Nasu-Hakola and healthy control subjects included in gene expression analyses of monocyte-derived macrophages

Subject ID	TREM2 genotype	Gender	Age	References
NHD1	C97T; homozygous	F	46	[4]
NHD2	C97T; homozygous	M	47	[4]
NHD3	482 + 2T → C; homozygous	F	53	[39]
CTR1	Normal TREM2 alleles	F	60	[4]
CTR2	Normal TREM2 alleles	M	38	NA
CTR3	Normal TREM2 alleles	M	38	NA

Age was at time of analysis

*NHD* Nasu–Hakola disease, *CTR* control/healthy subjects, *NA* not applicable

### Microarray processing and analysis

In order to analyze the Affymetrix HuGene-1_0-st-v1 microarrays, CEL files were uploaded into Thermo Fisher’s Transcriptome Analysis Console (TAC) Software. We normalized the data in TAC and did differential expression analysis to compare gene expression in 3 control samples with 3 samples carrying TREM2 homozygous mutations. The differentially expressed genes were graphed in a volcano plot, with the − log10 of the *P* value on the *Y*-axis and the fold change on the *X*-axis. A twofold or greater change in gene expression and a *P* value of 0.05 were considered significant and colored red for up-regulated genes and green for down-regulated genes.

### Statistical analysis

Data are displayed as individual dots and mean ± SEM. For each graph, the number of observations indicated with ‘‘*n*’’ and the number of biological replicates (mice) indicated with ‘‘*N*’’ can be found in the figure legends. Differences between multiple groups were analyzed by one-way ANOVA and a post hoc test (Tukey’s or Sidak’s or Dunnett’s). Comparisons between two groups following a normal distribution were analyzed using an unpaired *t* test (two-tail distribution) or a Mann–Whitney test when the distribution was not parametric, as indicated in each figure. Statistical analysis was performed using GraphPad Prism (Graph-Pad Software). Values were considered significant if *P* < 0.05. 

## Results

### TREM2 is highly expressed in active MS lesions

**Fig. 1:**
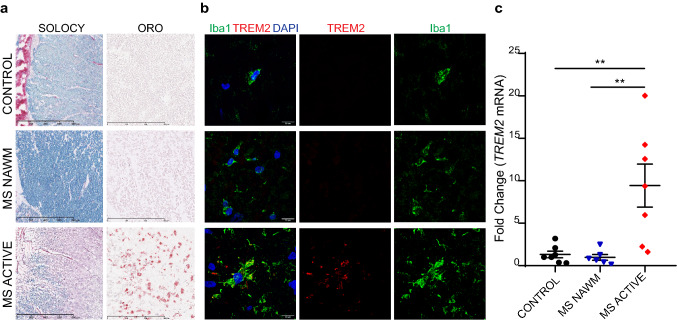
TREM2 is highly expressed in active MS lesions. **a** Top to bottom: white matter with no pathology in control non-MS individuals (CONTROL), normal appearing white matter of MS subjects (MS NAWM) and MS active lesions in the white matter (MS ACTIVE) are stained with solochrome cyanine staining (SOLOCY) and with Oil Red O staining (ORO). Original magnification SOLOCY: × 10; scale bar: 800 µm; ORO: × 20; scale bar: 300 µm. **b** Representative confocal images of Iba1 (green), TREM2 (red), and DAPI (blue) in CONTROL, MS NAWM, and MS ACTIVE lesions. Original magnification, × 63. Scale bar, 10 µm. **c** Relative *TREM2* mRNA expression by qPCR of the previously described tissue types. 7 samples from 4 CONTROLS, 6 NAWM samples from 3 MS patients, and 7 ACTIVE lesion samples from 5 MS patients. **P < 0.01, One-way ANOVA with Tukey’s post hoc test

The general consensus is that TREM2 is expressed by microglia in the CNS, especially after activation [6, 21, 47]. TREM2 expression was reported in active demyelinating lesions on myelin-laden macrophages (called foamy macrophages), which are clearing out myelin debris, a critical step to allow remyelination [41]. Yet, a systematic characterization of TREM2 expression on microglia-macrophages in different types of MS lesions has not been done. Here we studied TREM2 expression by immunohistochemistry (IHC) and real-time qPCR in human CNS tissues derived from autopsy of subjects with MS or unaffected controls (Table 1). We specifically analyzed MS active lesions and normal appearing white matter (NAWM) in MS subjects and white matter with no pathology in non-MS individuals as control. By confocal analysis we showed TREM2 specific expression in ionized calcium-binding adapter molecule 1(Iba1)^+^ cells in active MS lesion, which were identified as oil-red O^+^ lipid laden macrophages-microglia by histology (Fig. 1a, b). TREM2 was not detectable by IHC on Iba1^+^ microglia in NAWM in MS subjects as well as in the white matter of control individuals (Fig. 1b).

To quantify *TREM2* expression in different lesion types, we performed quantitative real-time RT-PCR (RT-qPCR) studies which demonstrated that *TREM2* expression is significantly higher in active MS lesions compared to MS NAWM or white matter from controls (Fig. 1c).

These results provide evidence that TREM2 is highly expressed by Iba1^+^ cells present in active demyelinating MS lesions. Therefore, we sought to further evaluate TREM2 potential involvement in the context of CNS demyelination and myelin debris clearance by microglia.

### Effects of a newly generated TREM2-specific antibody on TREM2 signaling

**Fig. 2:**
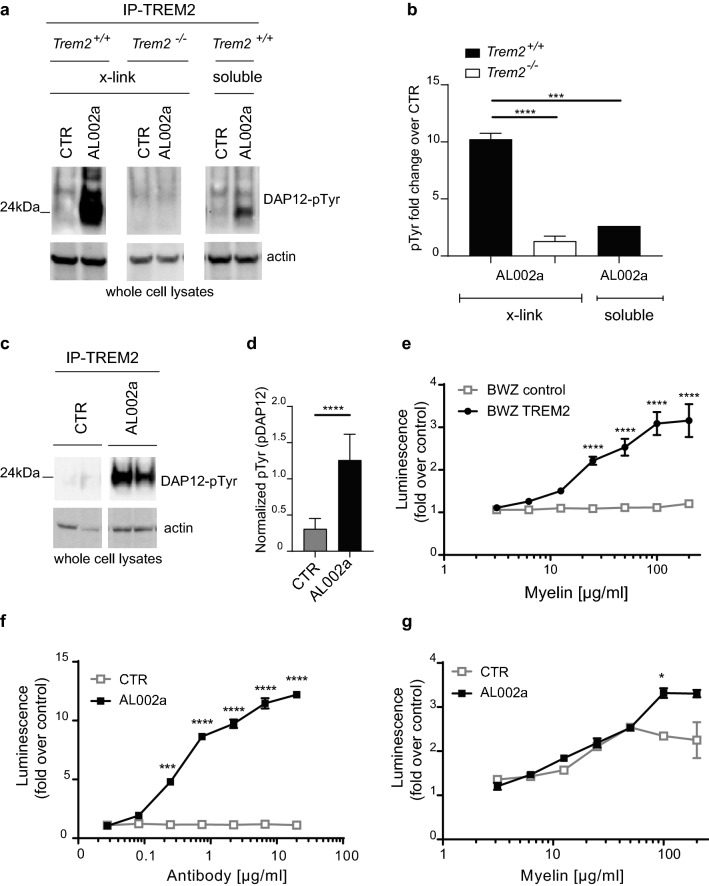
In vitro and in vivo effects of a TREM2-specific antibody on TREM2 signaling. **a** WB analysis of *Trem2*^+*/*+^ or *Trem2*^−*/*−^ BMDM stimulated with a TREM2-specific antibody (AL002a) or with a control antibody, by cross-linking with a secondary antibody (x-link) or in solution (soluble; for *Trem2*^+*/*+^ only). Cells were immunoprecipitated with a rat anti-h/m-TREM2 antibody and fractionated by SDS-PAGE in non-reducing conditions. Phopho DAP12 (pTyr) is shown here as a dimer. **b** Quantification of DAP12-pTyr normalized on actin is shown as fold change over control. ***P < 0.001, ****P < 0.0001, One-way ANOVA with Tukey’s post hoc test. **c** Peritoneal cells isolated from *Trem2*^+*/*+^ mice after thyoglicollate treatment, immunoprecipitated with an anti-TREM2 antibody (clone 237920), and analyzed by WB. **d** Quantification of DAP12- pTyr normalized on actin. ****P < 0.0001, two-tailed unpaired Student’s *t* test. **e** Activation of TREM2 signaling in the BWZ reporter cell assay with different concentrations of plate-bound myelin. BWZ cells express either NFAT:luciferase alone (BWZ control) or in combination with mouse TREM2 and DAP12 (BWZ TREM2). ****P < 0.0001, Two-way ANOVA with Sidak’s post hoc test. **f** TREM2 signaling in the BWZ *Trem2* reporter cell assay at different concentrations of AL002a antibody. ***P < 0.001, ****P < 0.0001, Two-way ANOVA with Dunnett’s post hoc test. **g** Myelin-induced signaling by AL002a (10 µg/ml) in the BWZ *Trem2* reporter cell assay at different concentrations of plate-bound myelin. *P < 0.05, Two-way ANOVA with Dunnett’s post hoc test. *CTR* control

To characterize TREM2 functions on microglia during CNS demyelination, we generated a monoclonal antibody targeting mouse TREM2, called AL002a. The antibody’s specificity was verified by binding to wild-type (*Trem2*^+/+^), but not *Trem2*^−*/*−^ thioglycollate-induced macrophages (THG-Mac) (Supplementary Fig. 1a). To test whether AL002a induced signaling after receptor engagement, *Trem2*^+*/*+^ and *Trem2*^−*/*−^ BMDM were stimulated in vitro with AL002a, followed by crosslinking with a secondary antibody to induce receptor clustering. AL002a induced phosphorylation of the TREM2-associated adaptor protein DAP12 in *Trem2*^+*/*+^, but not in *Trem2*^−*/*−^ BMDM (Fig. 2a, b; DAP12 phosphorylation is indicated as DAP12-pTyr). Treatment with an isotype control antibody did not show any DAP12 activation. DAP12 phosphorylation was also induced when BMDM were stimulated with AL002a without secondary cross-linking (soluble) (Fig. 2a, b). These results demonstrate that AL002a is a strong inducer of DAP12 phosphorylation (after cross-linking and as soluble), and it acts as an agonist of TREM2 signaling.

Next, we investigated whether AL002a was able to induce TREM2 clustering and signaling activation in vivo. Mice were injected with the anti-TREM2 antibody after thioglycollate-induced recruitment of macrophages to the peritoneal cavity. AL002a induced phosphorylation of DAP12-associated TREM2 compared to the isotype control (Fig. 2c, d). These results demonstrate that AL002a is a potent inducer of TREM2 intracellular signaling in vitro (by both cross-linking and soluble stimulation) and in vivo (by soluble stimulation).

It was previously demonstrated that TREM2 recognizes myelin-associated lipids [42, 52], suggesting that TREM2 may directly sense lipid components exposed after myelin damage. To confirm these results, we developed a cell reporter assay, using BWZ cells expressing the mouse TREM2/DAP12 complex and transduced with a luciferase reporter gene under NFAT promoter control. TREM2 clustering promotes calcium flux stimulating NFAT-activity that induces luciferase expression [50]. In the presence of purified human myelin, TREM2 signaling was activated in the BWZ cell reporter system in a dose-dependent fashion (Fig. 2e). Next, we tested the ability of soluble AL002a to activate TREM2 signaling in the reporter cell assay. AL002a induced TREM2 signaling as demonstrated by incremental luciferase induction in a dose-dependent manner (Fig. 2f). Subsequently, we tested whether TREM2 antibodies could block or enhance myelin-induced TREM2 signaling. To this end, we added AL002a to BWZ cells seeded on increasing concentrations of plate-bound myelin. At lower myelin concentrations (< 100 µg/ml), AL002a antibody did not modulate signaling; in contrast, at higher concentrations (> 100 µg/ml), AL002a significantly increased myelin-induced luciferase activation (Fig. 2g). These results show that AL002a potentiates myelin-mediated TREM2 signaling activation in vitro.

In conclusion, our data demonstrate that AL002a acts as a potent TREM2 agonist both in vitro and in vivo, and its agonistic activity is boosted by the presence of myelin.

### *Trem2* haploinsufficiency results in defective myelin debris clearance in the CPZ model of CNS demyelination

**Fig. 3:**
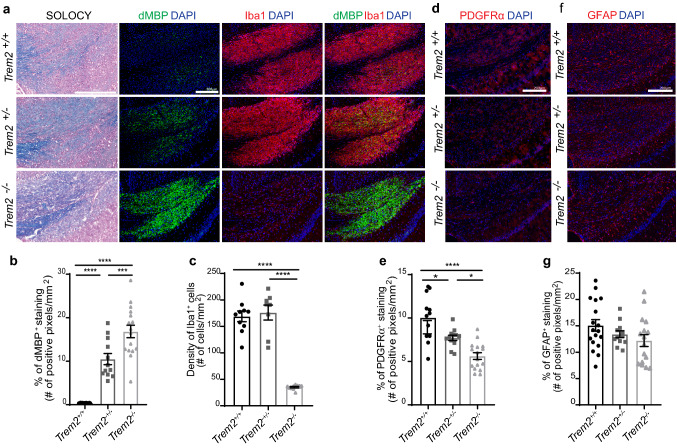
Trem2^+/-^ mice show defective myelin debris clearence after CPZ-induced demyelination. **a** Representative images of the corpus callosum (CC) of *Trem2*^+*/*+^, *Trem2*^+*/*−^ and *Trem2*^−*/*−^ mice fed with cuprizone (CPZ) for 4 weeks and then analyzed. Myelin was studied histologically by solochrome cyanine (SOLOCY) staining (intact myelin is stained in dark blue). Scale bar, 300 µm. dMBP (green) was used to assess myelin debris; microglia were analyzed as density of Iba1^+^ cells (red); DAPI (blue). Original magnification, × 10. Scale bar, 200 µm. Quantification of **b** dMBP fluorescent staining and **c** microglia density in the CC after 4 weeks on CPZ. dMBP quantification: *Trem2*^+*/*+^ *N* = 8 mice, *n* = 16 fields; *Trem2*^+*/*−^ *N* = 6, *n* = 12; *Trem2*^−*/*−^ *N* = 8, *n* = 16. Iba1 quantification: *Trem2*^+*/*+^ *N* = 5, *n* = 10; *Trem2*^+*/*−^ *N* = 4, *n* = 8; *Trem2*^−*/*−^ *N* = 5, *n* = 10. ***P < 0.001, ****P < 0.0001, One-way ANOVA with Tukey’s post hoc test. Representative images of PDGFRα (red) (**d**) and GFAP (red) (**f**) in the CC of *Trem2*^+*/*+^, *Trem2*^+*/*−^ and *Trem2*^−*/*−^ mice after 4 weeks on CPZ. Original magnification, × 10. Scale bar, 200 µm. Quantification of **e** PDGFRα and **g** GFAP fluorescent staining in the CC after 4 weeks on CPZ. PDGFRα quantification: *Trem2*^+*/*+^ *N* = 6, *n* = 12; *Trem2*^+*/*−^ *N* = 6, *n* = 12; *Trem2*^−*/*−^ *N* = 8, *n* = 16. GFAP quantification: *Trem2*^+*/*+^ *N* = 9, *n* = 18; *Trem2*^+*/*−^ *N* = 6, *n* = 12; *Trem2*^−*/*−^ *N* = 8, *n* = 16. *P < 0.05, ****P < 0.0001, One-way ANOVA with Tukey’s post hoc test

We and others have previously shown that *Trem2*^−*/*−^ mice present a significant defect in clearance of myelin debris in the CPZ-induced model of demyelination compared to *Trem2*^+*/*+^ mice [8, 42]. Whether heterozygous (*Trem2*^+*/−*^) mice would present the same or a milder phenotype than *Trem2*^−*/*−^ mice has not been evaluated before in the CPZ model. Here, *Trem2*^+/+^, *Trem2*^+*/−*^, and *Trem2*^−*/*−^ mice were fed with CPZ for 4 weeks to induce CNS demyelination and to evaluate microglia response to the injury, the degree of myelin debris clearance and remyelination. We performed IHC analyses within the corpus callosum (CC) (Supplementary Fig. 1b), a site of particularly profound and consistent demyelination in the CPZ model [31]. Solochrome cyanine staining was performed to qualitatively visualize myelin in the CC (Fig. 3a). Intact myelin was colored in dark blue in naïve mice (Supplementary Fig. 1c). In contrast, a light blue myelin staining was evident in the CC in *Trem2*^+*/−*^, and *Trem2*^−*/*−^ mice fed CPZ. This was suggestive of tissue accumulation of myelin debris, as we have observed in our previous study [8]. To assess the amount of myelin debris accumulation in the tissue we detected degraded myelin basic protein (dMBP) by using an antibody able to bind MBP epitopes exposed only after myelin degradation [30]. As previously reported, when compared to *Trem2*^+*/*+^, *Trem2*^−*/*−^ mice after 4 weeks on CPZ displayed a significant increase in dMBP signal, accompanied by a significant reduction in Iba1^+^ microglia [8] (Fig. 3a–c) and in OPC numbers, identified as Platelet-Derived Growth Factor Receptor Alpha (PDGFRα)^+^ cells (Fig. 3d, e). No differences were found in astrocyte activation, quantified as glial fibrillary acidic protein (GFAP) positive cells (Fig. 3f, g). Notably, *Trem2*^+*/−*^ mice displayed a phenotype which was intermediate between *Trem2*^+*/*+^ and *Trem2*^−*/*−^ mice showing a significant increase in dMBP (Fig. 3a, b) and significantly less PDGFRα^+^ OPCs compared to *Trem2*^+*/*+^ mice (Fig. 3d, e). No differences were noted in the number of Iba1^+^ nor GFAP^+^ cells in the CC in *Trem2*^+*/−*^ compared to *Trem2*^+*/*+^ mice (Fig. 3a, c, f, g), in contrast to the significant reduction of the microglia number observed in *Trem2*^−*/*−^ vs. *Trem2*^+*/*+^. These results suggest that the presence of a fully functional TREM2 receptor on microglia is critical for efficient myelin debris removal and quick OPC recruitment observed in *Trem2*^+*/*+^ mice. *Trem2* haploinsufficiency leads to inefficient myelin debris clearance, reduced OPC recruitment, even if it does not impact on the number of microglia accumulating in the CC.

### Treatment of *Trem2*^+*/−*^ mice with AL002a antibody accelerates myelin debris clearance

**Fig. 4:**
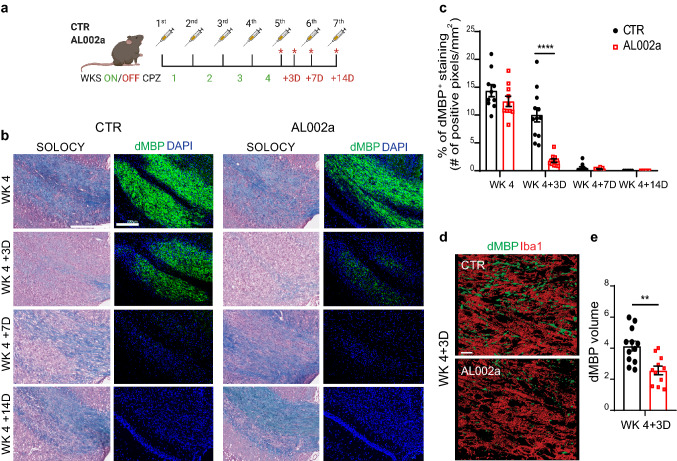
Treatment with the AL002a antibody during CPZ-induced CNS demyelination in *Trem2*^+*/*−^ mice promotes myelin debris clearance in the corpus callosum. **a** Timeline of antibody treatment during cuprizone experiment. Weeks on CPZ = WKS ON (green numbers); days after CPZ withdrawal = OFF (red numbers). Mice were sacrificed after 4 weeks on CPZ (WK 4), 4 weeks on CPZ followed by 3 days (+ 3D), 7 days (+ 7D) or 14 days (+ 14D) of recovery (red asterisks correspond to the time points in which the tissues were collected). **b** Representative images of solochrome cyanine (SOLOCY) staining and of dMBP (green) and DAPI (blue) immunostaining in the CC of *Trem2*^+*/*−^ mice at the different time points. Original magnification, × 10. Scale bar, SOLOCY, 300 µm; dMBP, 200 µm. **c** Quantification of dMBP debris at the different time points. CTR group: WK 4 *N* = 5 mice, *n* = 10 fields; WK 4 + 3D *N* = 6, *n* = 12; WK 4 + 7D *N* = 5, *n* = 10; WK 4 + 14D *N* = 5, *n* = 5; AL002a-treated group: WK 4 *N* = 5, *n* = 10; WK 4 + 3D *N* = 5, *n* = 10; WK 4 + 7D *N* = 4, *n* = 8; WK 4 + 14D *N* = 6, *n* = 6. ****P < 0.0001, two-tailed unpaired Student’s *t* test. **d** 3D reconstruction by Imaris of Iba1^+^ cells (red) and dMBP (green) immunostaining in the CC at WK 4 + 3D. Original magnification, × 63. Scale bar, 10 µm. **e** Quantification of dMBP total volume per field in mice treated with AL002a or CTR. CTR group: *N* = 6, *n* = 12; AL002a treated group: *N* = 6, *n* = 11. **P < 0.01, two-tailed unpaired Student’s *t* test

Next, we assessed the potential impact of AL002a on myelin debris clearance during CPZ-induced demyelination in *Trem2*^+*/−*^ mice. To this end, *Trem2*^+*/−*^ mice were treated with AL002a or an isotype control given intraperitoneally 4 days before starting CPZ feeding and then weekly throughout the experiment (Fig. 4a). AL002a was able to reach the brain and it was detected in the CC and in the cortex (Ctx) (Supplementary Fig. 1d). Analyses were performed at 4 weeks (WK 4; when the peak of demyelination is observed) after CPZ feeding and then at 3 days (WK 4 + 3D), 7 days (WK 4 + 7D), and 14 days (WK 4 + 14D) after CPZ withdrawal, when mice were fed regular chow to allow remyelination. Treatment in vivo with AL002a resulted in the amelioration of CPZ-induced pathology in *Trem2*^+*/−*^ mice. Specifically, AL002a treatment enhanced myelin debris clearance compared to *Trem2*^+*/−*^ mice treated with the control antibody, as demonstrated by a significant reduction in dMBP staining at WK 4 + 3D after CPZ withdrawal (Fig. 4b, c). This finding was also confirmed by confocal microscopy and 3D-reconstruction, which showed a significant reduction of dMBP amount (quantified as dMBP volume) per field at the same timepoint in AL002a versus control-treated *Trem2*^+*/−*^ mice (Fig. 4d, e). Since TREM2 receptor is specifically expressed by microglia in the brain, these results strongly suggest that AL002a antibody accelerates myelin clearance in the CPZ model of demyelination by potentiating TREM2 receptor function on microglial cells.

### AL002a antibody promotes myelin uptake and degradation in vivo and in vitro

**Fig. 5:**
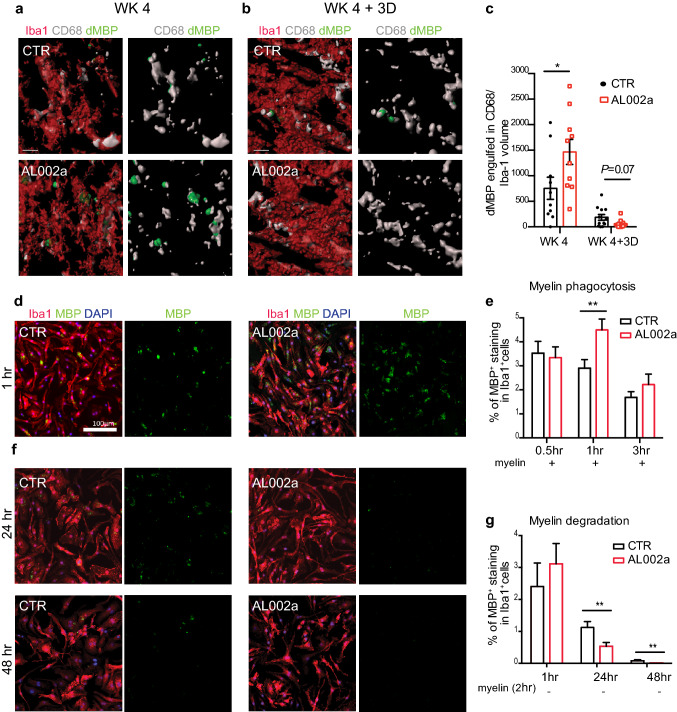
AL002a treatment enhances myelin uptake and processing by *Trem2*^+*/*−^ microglia in vivo and by BMDM in vitro. Representative confocal z-stack and relative 3D surface rendering showing volume reconstruction of microglia (red), CD68 (gray) and dMBP (green), detected within microglial CD68^+^ structures at WK 4 (**a**) and WK 4 + 3D (**b**) in the CC of *Trem2*^+*/*−^ mice. Original magnification, × 63. Scale bar, 3 µm. **c** Quantification of engulfed dMBP within CD68 per microglia at WK 4 and WK 4 + 3D. CTR group: WK 4 *N* = 5 mice, *n* = 10 fields; WK 4 + 3D *N* = 6, *n* = 12. AL002a-treated group: WK 4 *N* = 5, *n* = 10; WK 4 + 3D *N* = 6, *n* = 11; *P < 0.05, two-tailed unpaired Student’s *t* test. **d** Representative images and **e** relative quantification of *Trem2*^+*/*−^ BMDM pre-stimulated in vitro with AL002a and control antibodies (10 µg/ml) and treated with myelin for 0.5, 1 and 3 h. Iba1 (red), MBP (green), DAPI (blue). Original magnification, × 10. Scale bar, 100 µm. CTR group: 30’ *n* = 80; 1 h *n* = 100; 3 h *n* = 80; AL002a group: 30’ *n* = 80; 1 h *n* = 100; 3 h *n* = 80. **P < 0.01, two-tailed unpaired Student’s *t* test. **f** Representative images and **g** relative quantification of *Trem2*^+*/*−^ BMDM assayed for their degradative capacity. *Trem2*^+*/*−^ BMDM were pre-stimulated with AL002a or control antibodies (10 µg/ml), then incubated with human myelin for 2 h and washed. MBP content in Iba1^+^ cells was quantified after 1, 24, and 48 h. CTR group: 1 h *n* = 42; 24 h *n* = 104; 48 h *n* = 80; AL002a group: 1 h *n* = 48; 24 h *n* = 110; 48 h *n* = 80. **P < 0.01, two-tailed unpaired Student’s *t* test. **d**–**g** Three independent experiments were performed

Microglia play a key role in myelin debris removal after CNS demyelination [33]. Microglia internalize and phagocytose myelin which is then degraded intracellularly by lysosomal structures. We hypothesized that reduction in dMBP staining observed after AL002a treatment could be related to an increased ability to uptake and/or to degrade myelin fragments by *Trem2*^+*/−*^ microglia. To clarify these aspects, the amount of dMBP within microglial phagolysosomes was analyzed by immunofluorescence in the CC at WK 4 and WK 4 + 3D. Quantitative confocal analysis and 3D reconstruction of Iba1^+^ cells showed a significant increase in dMBP amount inside CD68^+^ phagolysosomal structures at WK 4 in AL002a vs. control treated mice (Fig. 5a, c). Interestingly, 3 days after toxin withdrawal we observed that dMBP in phagolysosomes was lower in AL002a-treated mice compared to control (Fig. 5b, c; *P* = 0.07). These findings suggest that AL002a antibody treatment enhanced myelin debris internalization by microglia at WK 4, as demonstrated by a higher dMBP amount detected inside CD68^+^ structures in microglia (Fig. 5a, c), accompanied by decreased dMBP total volume per field (Fig. 4b, c) at WK 4 + 3D. Moreover, the reduction in dMBP content within lysosomal structures after 3 days from toxin withdrawal implies that AL002a further accelerated intracellular myelin processing and degradation.

To confirm that AL002a antibody was improving myelin uptake and degradation capacity by microglia, we performed an in vitro myelin phagocytic and degradation assay. To assess myelin phagocytosis, BMDMs from *Trem2*^+*/−*^ mice were pre-treated with AL002a or control antibody and then myelin was added to the culture. Analysis of MBP internalization by immunofluorescence revealed a significant increase in MBP amount detected inside Iba1^+^ cells treated with AL002a after 1 h (Fig. 5d, e), while no differences between the two groups were detected at 30’ and 3 h. This suggests a time-dependent increase in myelin uptake by BMDM after AL002a treatment and further supports our in vivo findings showing higher dMBP amount inside microglia at WK 4 on CPZ in AL002 treated group (Fig. 5a, c).

To assess the effect of AL002a on myelin degradation, *Trem2*^+*/−*^ BMDM were pre-treated with AL002 or control antibodies, then incubated with human myelin for 2 h, and subsequently washed in order to remove extracellular myelin not taken up. MBP content by BMDM was then quantified after 1, 24, and 48 h. We observed a significant reduction in myelin content in BMDM treated with AL002a compared to those treated with the control antibody both at 24 and 48 h (Fig. 5f, g). Therefore, AL002a leads to a significant increase in myelin phagocytosis by *Trem2*^+*/−*^ BMDM after 1 h, and also it accelerates their degradative capacity at later time points. These findings are consistent with the observation that AL002a treatment promotes a more efficient clearance of myelin debris by *Trem2*^+*/−*^ microglia in vivo in the CPZ model.

### Treatment with AL002a enhances the activation state of Trem2^+*/−*^ microglia

AL002a could potentially promote myelin debris clearance by *Trem2*^+*/−*^ microglia after CPZ by increasing microglia numbers in the areas of demyelination and/or their phagocytic functions. Since we did not observe any difference in microglia density (Supplementary Fig. 2a and b) nor proliferation (the latter quantified as Iba1^+^BrdU^+^ cells at WK 4 + 3D; Supplementary Fig. 2c and d) in *Trem2*^+*/−*^ mice after treatment with AL002a, we hypothesized that AL002a antibody could enhance microglia activation state as well as phagocytic and lysosomal activity.

**Fig. 6:**
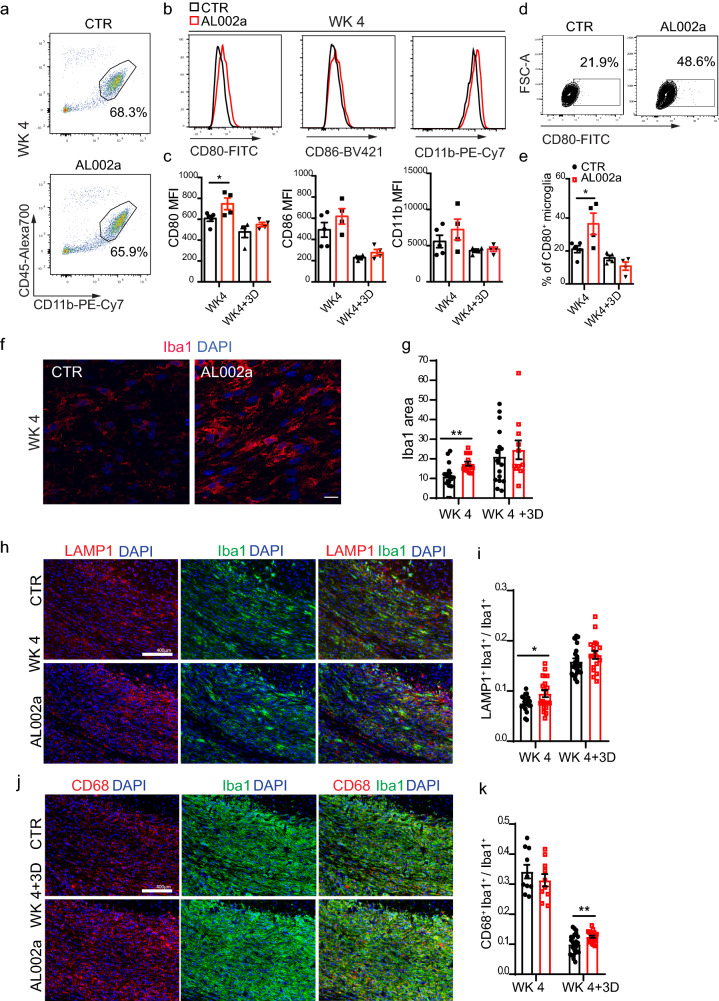
Increased *Trem2*^+*/*−^ microglia activation following AL002a antibody treatment in vivo. **a** FACS analysis of *Trem2*^+*/*−^ microglia isolated from the CC at WK 4. Microglia were identified as CD11b^+^CD45^int^ cells upon doublet discrimination and death cell exclusion. Values indicated in FACS plots represent the relative frequency of live cells after duplets exclusion. **b** Histograms showing the mean fluorescence intensities (MFIs) of surface CD80, CD86, and CD11b at WK 4 and **c** their relative quantification at WK 4 and WK 4 + 3D. **d** Example plots of flow cytometric analysis of CD80 positive microglia at WK 4 and **e** quantification of the microglia percentage expressing CD80 molecule at WK 4 and WK 4 + 3D. CTR group *N* = 5 and AL002a treated group *N* = 4; *P < 0.05, two-tailed unpaired Student’s *t* test. **f** Representative confocal images of Iba1 (red) and DAPI (blue) in the CC at WK 4. Original magnification, × 63. Scale bar, 10 µm. **g** Quantification of Iba1 intensity (measured as area) at WK 4 and WK 4 + 3D. CTR group: WK 4 *N* = 5 mice, *n* = 15 fields; WK 4 + 3D *N* = 6, *n* = 16; AL002a-treated group: WK 4 *N* = 5, *n* = 15; WK 4 + 3D *N* = 6, *n* = 11; **P < 0.01 two-tailed unpaired Student’s *t* test. **h** Representative images of LAMP1 (red), Iba1(green) and DAPI (blue) at WK 4. Original magnification, × 20. Scale bar, 400 µm. **i** Quantification of LAMP1^+^ signal colocalizing with Iba1^+^ cells. CTR group: WK 4 *N* = 5, *n* = 20; WK 4 + 3D *N* = 6, *n* = 23; AL002a treated group: WK 4 *N* = 5, *n* = 20; WK 4 + 3D *N* = 5, *n* = 18; *P < 0.05, two-tailed unpaired Student’s *t* test. **j** Representative images of CD68 (red), Iba1 (green) and DAPI (blue) at WK 4. Original magnification, × 20. Scale bar, 400 µm. **k** Quantification of CD68^+^ signal colocalizing with Iba1^+^ cells. CTR group: WK 4 *N* = 5, *n* = 10; WK 4 + 3D *N* = 6, *n* = 24; AL002a-treated group: WK 4 *N* = 5, *n* = 10; WK 4 + 3D *N* = 5, *n* = 20; **P < 0.01 two-tailed unpaired Student’s *t* test

FACS analysis of CD11b^+^ CD45^int^ microglia isolated from the CC (Fig. 6a and Supplementary Fig. 3a) showed a significant increase in CD80 costimulatory molecule at WK 4 measured both as mean fluorescence intensity (MFI) and as cell percentage in AL002a treated vs. control (Fig. 6b, c). No differences were observed in CD86 and CD11b expression on microglia (Fig. 6b, c). Iba1 intensity in the CC, detected by immunofluorescence, was also higher in AL002a-treated compared to the control group at WK 4 (Fig. 6f, g), thus confirming the increased activation of microglia after AL002a treatment. At 3 days after CPZ withdrawal, there were no differences in microglia activation state between the two groups (Fig. 6c–e), thus indicating that CPZ removal was sufficient to revert AL002a effects on microglia inflammatory state. A strong upregulation of activation markers after AL002a treatment was also detectable in microglia isolated from the hippocampus (HP) at WK 4 (Supplementary Fig. 3b–g), an area which is also affected by pathology after CPZ administration [15]. This result further corroborates AL002a effect in promoting microglia activation in the CPZ model of demyelination.

Additionally, at WK 4 after CPZ, the lysosomal-associated membrane protein 1 (LAMP1), a protein essential for the fusion of phagosomes with lysosomes and their subsequent degradation [12], was significantly increased in microglia from AL002a-treated mice compared to controls (Fig. 6h, i). WK 4 represents the peak of demyelination and microglia are actively phagocytosing myelin debris at this time. Notably, we also detected a significant increase in CD68^+^ phagosome structures in *Trem2*^+*/−*^ microglia at WK 4 + 3D compared to control (Fig. 6j, k). This further supports enhanced in vivo phagocytic activities by microglia following AL002a-induced activation.

### AL002a treatment leads to increased OPC density, OL maturation, and remyelination

**Fig. 7:**
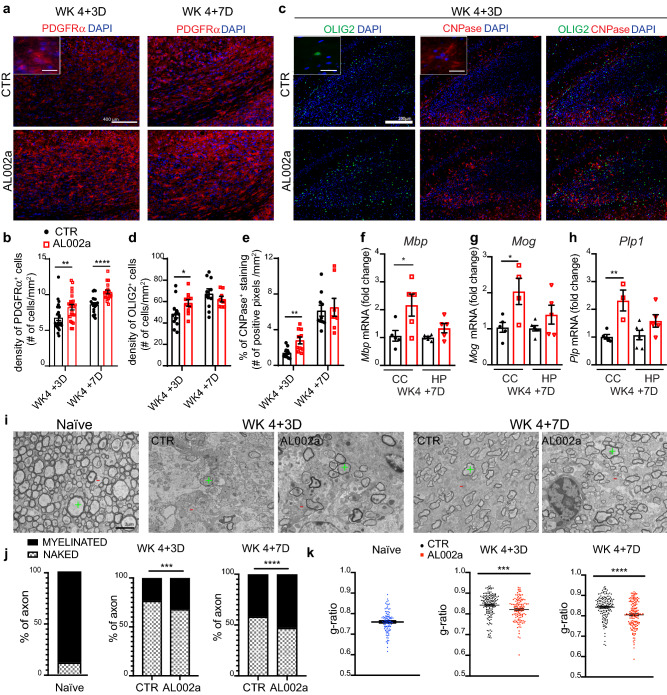
AL002a -treated mice show increased OPC density, mature myelin and remyelination after CPZ-induced demyelination. **a** Representative images of PDGFRα^+^ (red) and DAPI (blue) in the CC at WK 4 + 3D and WK 4 + 7D. Original magnification, × 20. Scale bar, 400 µm. Insert magnification, × 60. Scale bar, 25 µm. **b** Quantification of PDGFRα^+^ cell density. CTR group: WK 4 + 3D *N* = 6 mice, *n* = 24 fields; WK 4 + 7D *N* = 5, *n* = 20; AL002a-treated group: WK 4 + 3D *N* = 6, *n* = 24; WK 4 + 7D *N* = 5, *n* = 20; **P ≤ 0.01; ****P ≤ 0.0001; WK 4 + 3D two-tailed Mann–Whitney test; WK 4 + 7D two-tailed unpaired Student’s *t* test. **c** Representative images of OLIG2 (green), CNPase (red) and DAPI (blue) in the CC at WK 4 + 3Ds and WK 4 + 7D. Original magnification, × 10. Scale bar, 200 µm. Insert magnification, × 60. Scale bar, 25 µm. **d** Quantification of the density of OLIG2^+^ cells at both the time points. **e** Quantification of the percentage of CNPase^+^ staining in the CC. CTR group: WK 4 + 3D *N* = 6, *n* = 12; WK 4 + 7D *N* = 5, *n* = 10; AL002a-treated group: WK 4 + 3D *N* = 5, *n* = 10; WK 4 + 7D *N* = 4, *n* = 8; *P < 0.05, **P ≤ 0.01; two-tailed unpaired Student’s *t* test. RT-qPCR quantification of mRNA levels of myelin basic protein (*Mbp*) (**f**), myelin oligodendrocyte glycoprotein (*Mog*) (**g**) and proteolipid protein 1 (*Plp1*) (**h**) in the corpus callosum (CC) and the hippocampus (HP) of *Trem2*^+*/*−^ mice treated with AL002a antibody or CTR. Tissues were collected at WK 4 + 7D. CTR group: *N* = 5, AL002a-treated group: *N* = 5 (*Mpb*); *N* = 4 (*Mog*); *N* = 3 (*Plp1*). *P < 0.05, two-tailed unpaired Student’s *t* test. **i** Electron microscopy (EM) of a naïve mouse and mice treated with AL002a or isotype control antibody at WK 4 + 3D and WK 4 + 7D. Green plus sign indicates myelinated axons and red minus sign indicates naked axons. Original image = × 5000. Scale bar = 2 µm. **j** Percentage of myelinated axons (MA) and naked axons (NA) in naïve mice, and in CTR and AL200a-treated mice at WK 4 +3D and WK 4 + 7D. ***P < 0.001 at WK 4 + 3D and ****P < 0.0001 at WK 4 + 7D, two-tailed Mann–Whitney test. **k** G-ratio of myelinated fibers in naïve mice, and in CTR and AL002a-treated mice at WK 4 + 3D and WK 4 + 7D. Each dot represents one axon. Naïve mice *N* = 2, CTR treated mice at WK 4 + 3D *N* = 5 and at WK 4 + 7D *N* = 5; AL002a-treated mice at WK 4 + 3D *N* = 4 and at WK 4 + 7D *N* = 5. ***P < 0.001 at WK 4 + 3D and ****P < 0.0001 at WK 4 + 7D, two-tailed unpaired Student’s *t* test

Microglia activation and myelin debris clearance are critical steps for successful remyelination [29]. Therefore, we hypothesized that AL002a treatment, by promoting myelin debris clearance by microglia, could positively impact remyelination. We have previously shown that increased accumulation of damaged myelin in the CC of *Trem2*^+*/−*^ mice, compared to *Trem2*^+*/*+^ mice, was associated with a reduced number of PDGFRα^+^ OPCs (Fig. 3a, b, d, e). Efficient clearance of myelin debris after demyelination can favorably impact OPC recruitment and differentiation into remyelinating oligodendrocytes [29]. We observed a significant increase in PDGFRα^+^ OPC density in the CC of *Trem2*^+*/−*^ mice after AL002a antibody treatment at WK 4 + 3D and WK 4 + 7D compared to control (Fig. 7a, b). No differences in PDGFRα^+^ OPC proliferation, quantified as density of PDGFRα^+^BrdU^+^ cells, were observed at WK 4 + 3D (Supplementary Fig. 4a, b). These results suggest that AL002a antibody could promote the recruitment of OPCs from surrounding brain regions, rather than enhancing the proliferation of resident OPCs in the CC. We then analyzed the expression of OLIG2 and CNPase, markers specifically expressed by mature OL. We observed a significant increase of both OLIG2^+^ and CNPase^+^ cells upon AL002a treatment vs. control at WK 4 + 3D after CPZ withdrawal (Fig. 7c–e). The same increase in *Olig2* and *Cnp* genes was detected by RT-qPCR analyses at WK 4 +7D in both the CC and HP (Supplementary Fig. 4c and d). In parallel, we observed a significant increase in the mRNA of myelin genes such as myelin basic protein (*Mbp*), myelin-associated protein (*Mag*), and proteolipid protein 1 (*Plp1*) in both regions (Fig. 7f–h). Finally, to assess remyelination and thickness of the new myelin sheaths in the CC, we performed transmission electron microscopy (TEM) analysis on sagittal sections of *Trem2*^+*/−*^ mice naïve or fed with CPZ and analyzed after withdrawal at WK 4 + 3D and WK 4 + 7D (Fig. 7i). Specifically, myelinated axons (MA) and naked axons were counted in the CC of naïve mice (MA: 89% ± 0.00724 as mean ± SEM) or in mice fed with CPZ and treated with AL002a or control antibody. Significantly more MA were counted in the CC after AL002a treatment compared to control at WK 4 + 3D (MA: 32% ± 0.0192 in AL002a-treated group vs. MA: 23% ± 0.0122 in control group) and this was even more pronounced at WK 4 + 7D (MA: 53% ± 0.0124 in AL002a-treated group vs. MA:42% ± 0.0179 in control group) (Fig. 7j). In addition, g-ratio analysis revealed significantly increased myelin sheath thickness in mice treated with AL002a at WK 4 + 3D and WK 4 + 7D compared to control (Fig. 7k). All together, these data strongly suggest that activation of TREM2, through AL002a antibody, increased OPC accumulation within the CC lesioned area, enhanced OPC differentiation into mature oligodendrocyte, and accelerated remyelination.

### AL002a treatment preserves axonal integrity

**Fig. 8:**
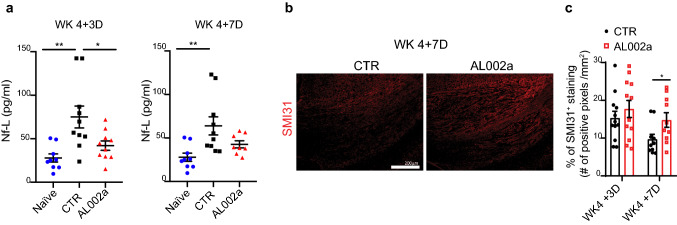
AL002a positively impacts axonal health after CPZ-induced demyelination. **a** Neurofilament light chain (Nf-L) levels were measured in plasma samples collected from *Trem2*^+*/*−^ naïve mice and *Trem2*^+*/*−^ mice treated with AL002a antibody or CTR at WK 4 +3D and WK 4 + 7D after CPZ-induced CNS demyelination. Naïve mice, *N* = 9; CTR group: WK 4 + 3D *N* = 10; WK 4 + 7D *N* = 10; AL002a-treated group: WK 4 + 3D *N* = 10; WK 4 + 7D *N* = 8. *P < 0.05, **P ≤ 0.01; one-way ANOVA with Tukey’s post hoc test. **b** Representative images and **c** quantification of SMI31 (red) fluorescent staining at WK 4 + 3D and WK 4 + 7D in the CC. CTR group: WK 4 + 3D *N* = 6 mice, *n* = 12 fields; 4 + 7D *N* = 5, *n* = 10; AL002a-treated group: WK 4 + 3D *N* = 6, *n* = 12; WK 4 + 7D *N* = 5, *n* = 10 fields; *P < 0.05; two-tailed unpaired Student’s *t* test

Remyelination is known to protect axons from ongoing degeneration [18]. Therefore, we further investigated whether AL002a together with promoting remyelination was also positively impacting axonal health. We assessed levels of neurofilament-light chain (Nf-L), which are released in significant quantity following axonal damage or neuronal degeneration, in *Trem2*^+*/−*^ mice treated with AL002a or control antibody. Mice treated with AL002a showed significantly lower Nf-L levels in the plasma at WK 4 + 3D compared to the control, with a trend of reduced levels also observed at WK 4 + 7D (Fig. 8a). These results are suggesting that AL002a treatment during CPZ-induced CNS demyelination will also preserve axonal integrity. Further evidence supporting a neuroprotective effect exerted by AL002a, came from the evaluation with the SMI-31 antibody (a marker of healthy axons) in the CC, which was significantly increased in AL002a compared to control treated *Trem2*^+*/−*^ mice at WK 4 + 7D (Fig. 8b, c). In conclusion, AL002a treatment preserved axonal health during CPZ treatment, thus preventing neurodegeneration.

### Macrophages derived from Nasu–Hakola subjects display a defect in phagocytic pathways

TREM2 function in human microglia is still elusive and this is also due to experimental difficulties and limitations of human studies. A role of TREM2 in phagocytic pathways in the human brain was previously suggested [13]. Here, we have analyzed the gene expression profile of macrophages obtained from the peripheral blood of three patients affected by NHD (lacking TREM2 receptor), compared to healthy control individuals. NHD is characterized by loss of myelin and microglia activation in the CNS [25]. These studies aimed to investigate whether a TREM2 loss of function mutation in NHD patients could result in the dysregulation of gene expression pathways involved in the clearance of myelin debris in the brain.

**Fig. 9:**
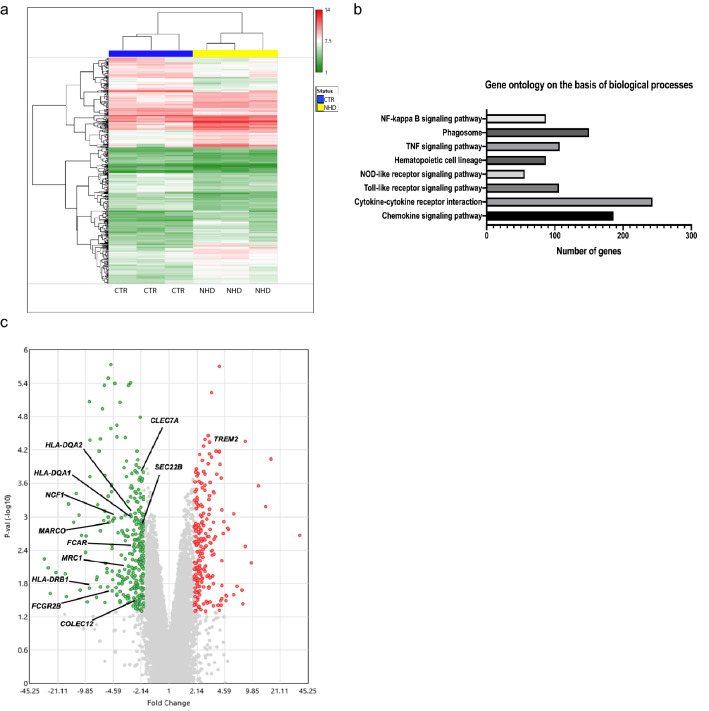
Phagocytic pathways dysregulation in macrophages derived from individuals affected by Nasu–Hakola disease. **a** Heat map showing different expression patterns of genes in Nasu–Hakola (NHD) patients and healthy controls (CTR). The heat map indicates up-regulation (red) and down-regulation (green). **b** Gene ontology analysis of the differentially expressed genes in NHD patients. **c** Volcano plots showing the distribution of gene expression fold changes in genes of the phagosome pathway. Genes with fold change > 2 and *P* value < 0.05 are indicated in red, and genes with fold change < − 2 and *P* value < 0.05 are indicated in green

The cohort of subjects that we studied was composed of: (1) two siblings affected by NHD and carrying an homozygous loss-of-function point mutation in exon 2 of the TREM2 gene (C97T) causing a glutamine in position 33 to be changed in a stop codon (Q33X) [4]; (2) one unaffected sibling from the same family, homozygous for the normal TREM2 allele, who was included as a control; (3) one NHD subject carrying a different homozygous mutation in the splice-donor consensus site of intron 3 of TREM2 (482 + 2T → C) [9]; (4) two unrelated healthy control subjects. Genetic and demographic characteristics are summarized in Table 2. Macrophages from each subject were differentiated in vitro from peripheral blood monocytes, and gene expression analysis was performed by microarray analysis. A total of 20,874 genes were tested to compare their expression between NHD subjects (*n* = 3) and unaffected controls (*n* = 3). 2526 genes were found to be differentially expressed with absolute fold change > 2 (ANOVA *P* value < 0.05). The heat map for these genes is displayed in Fig. 9a. Only 11 genes, including TREM2, remained significant after adjustment of the absolute fold change cutoff to a FDR < 0.05 [40] (Supplementary Table 1). Principal component analysis showed the ability of gene expression data to discriminate NHD from control subjects (Supplementary Fig. 4e). Gene ontology (GO) analysis revealed that genes involved in the phagosome pathway are among the top-pathways significantly altered in NHD subjects compared to controls along with changes in immune response pathways (Fig. 9b, and Supplementary Table 2 and 3). Interestingly, differentially expressed genes in the phagosome pathway were all downregulated in NHD vs. unaffected control subjects. The distribution of their fold changes and *P* values is represented in a volcano plot in Fig. 9c. Furthermore, we performed a co-expression analysis for TREM2 using public RNA-seq data from 31,499 samples and then compared these genes with the differentially expressed genes in NHD patients (Supplementary Table 4). A GO cellular component analysis confirmed that genes from the phagocytic pathway are both significantly co-expressed with TREM2 and altered in NHD patients (Supplementary Table 5). Overall, these data from NHD individuals, confirmed that TREM2 receptor plays a critical role in phagocytic pathways in human myeloid cells and that during NHD pathology genes involved in phagocytosis are specifically dysregulated.

## Discussion

This study provides evidence of beneficial effects of treatment with the new agonistic anti-TREM2 antibody AL002a in the CPZ model of CNS demyelination. We showed that TREM2 is highly expressed on microglia/macrophages phagocyting myelin in MS active lesions. In CPZ-induced demyelination we further validated previous findings that loss of TREM2 reduces myelin clearance and we showed that loss of one copy of TREM2 has an intermediate phenotype, suggesting that myelin removal by microglia is sensitive to TREM2 copy numbers. We then demonstrated that treatment with AL002a in vivo promotes efficient clearance of myelin debris, with increased myelin phagocytosis and intracellular degradation after CPZ-induce demyelination. This was accompanied by increased microglia activation and by a higher expression of proteins involved in phagocytic and degradative pathways. Most importantly, AL002a treatment increased OPC density and differentiation into mature OL to support remyelination, thus finally leading to axonal health preservation. Notably, macrophages from NHD individuals with loss-of-function homozygous TREM2 mutations showed a defect in the phagocytic pathways, suggesting that findings from mouse studies may translate to humans.

Growing evidences suggest the important role of TREM2 in microglia function. We previously showed that *Trem2*^−*/*−^ mice have a striking defect in microglia activation and myelin debris clearance in the CPZ model [8]. Similarly, lack of TREM2 in AD experimental models led to reduced microglia number, clustering and activation around CNS amyloid plaques accompanied by decreased plaque compaction and increased neuritic damage [50, 51, 53]. In line with these observations, TREM2 was demonstrated to mediate the switch from a homeostatic to a disease-associated microglia (DAM) phenotype [21]. TREM2 on microglia may also act as a lipid sensor capable of binding myelin debris with subsequent engulfment and clearance [42]. Our data demonstrate that AL002a is a potent TREM2 agonist antibody capable of activating TREM2 in vitro and in vivo and to potentiate myelin-induced activation of TREM2 signaling. We further characterized the role of AL002a in the in vivo model of CPZ-induced CNS demyelination. First, we tested AL002a in *Trem2*^+/+^ mice, without detecting any difference in damaged myelin accumulation, number of microglia or OPC in the CC compared to the control group at 4 weeks on CPZ (data not shown). These results were not unexpected given the rapid microglia response to damage, efficient clearance of myelin debris and subsequent remyelination after CPZ in wild-type mice [17]. Next, we tested AL002a in *Trem2*^+*/−*^ mice, which had less efficient clearance of damaged myelin compared to their wild-type counterparts, giving us the opportunity to unveil any effect of antibody-mediated TREM2 activation on CPZ-induced pathology. Indeed, AL002a treatment in vivo significantly enhanced myelin debris clearance in *Trem2*^+*/−*^ mice compared to the control *Trem2*^+*/−*^ group. This was likely due to a direct effect of AL002a on microglia, which consistently was shown to express TREM2 [45]. AL002a treatment in vivo by activating TREM2-dependent intracellular pathways led to more efficient myelin phagocytosis and degradation by microglia. This was accompanied by increased microglia expression of markers of activation (Iba1 and CD80) and phagolysosomal activity (CD68 and LAMP1). Similar effects were observed in BMDM in vitro. Our results clearly suggest that TREM2 plays a key role in myelin engulfment and intracellular processing by microglia. TREM2 has often been referred to as a phagocytic receptor as it was demonstrated to be involved in microglia phagocytosis of apoptotic neurons [46], myelin [8] and β-amyloid [24]. However, our current and previous findings indicate a more complex TREM2 function not only in myelin uptake, but also in promoting myelin debris degradation through the phagolysosomal pathway [8]. These findings were corroborated by the analysis performed in macrophages derived in vitro from NHD patients revealing that genes involved in the phagosome pathway are significantly altered in NHD subjects compared to healthy controls along with changes in immune response pathways. Therefore, myelin and axonal degeneration observed in NHD underscore a critical function played by microglia and TREM2 in myelin clearance, phagocytic functions, and maintenance of neuronal integrity.

We have previously reported that *Trem2*^−*/*−^ mice have a significant defect compared to *Trem2*^+/+^ in microglia numbers and proliferation at 4 weeks on CPZ and this could contribute to the defect in removing myelin debris from the tissue [8]. Interestingly, in the current study at this time point no differences in the number of microglia were detected between *Trem2*^+*/−*^ and *Trem2*^+/+^ mice, despite more myelin debris were accumulating in the CC of *Trem2*^+*/−*^. This could suggest that one copy of TREM2 is sufficient to sustain microglia expansion after CPZ-induced damage, but cannot support a fully functional response by microglia to clear the conspicuous amount of myelin debris derived from massive oligodendrocyte death. Alternatively, it is possible that a defect in microglia proliferation in *Trem2*^+*/−*^ mice is present, but at earlier time points (before 4 weeks) and in a very narrow time window. To this end, a very robust microglia activation and increase in density have been shown to start at 2–3 weeks after CPZ diet initiation, reaching a plateau at 4–5 weeks [15]. Future studies are needed to clarify if TREM2 activation could also directly enhance microglia proliferation in vivo.

TREM2 has been described to be intimately linked to microglia lipid metabolism [8, 42]. Lipids have been proposed as candidates for TREM2 ligands either as free molecules, complexed in myelin or in apolipoprotein particles [1, 2, 42, 50]. A recent report demonstrated that TREM2 is highly expressed in the adipose tissue by lipid-associated macrophages (LAM) where it drives gene expression programs involved in phagocytosis and lipid metabolism [19]. LAM cells in adipose tissue indeed expressed a highly similar gene profile as disease-associated microglia in AD with the exception of few tissue-specific genes [19, 21]. Chronic demyelination in vivo (12 weeks on CPZ) caused a robust accumulation of cholesteryl ester (CE) and oxidized CE in the *Trem2*^−*/*–^ brain, suggesting that TREM2 might be a crucial transcriptional regulator of cholesterol transport and lipid metabolism in microglia [35]. In support of a strong link between TREM2 and lipid metabolism are also the original reports describing NHD as a lipid storage disease due to a genetic enzymatic defect leading to lipid and cholesterol accumulation in the brain and bone cysts [37]. Therefore, it is attractive to suggest that a major role for TREM2 in microglia would be intracellular processing and degradation of lipid-rich material (e.g. myelin, cell membranes), especially after extensive demyelination. In our previous work, we provided initial evidence to support this hypothesis by showing that TREM2 is not a limiting factor in myelin engulfment, but it was clearly essential for microglia capacity to degrade myelin during chronic demyelination [8]. In the current study, we further support this hypothesis and we expand by showing that AL002a treatment significantly increases microglia capacity to phagocyte and degrade myelin in vivo and in vitro. These results confirm TREM2 as a key regulator of phagocytic clearance of myelin debris and lipid metabolism by microglia.

The identification of therapies promoting remyelination is a new frontier to overcome in MS. Approved disease-modifying treatments for MS are targeting the inflammatory disease component by reducing attack frequency and severity. Currently, no therapies are available to regenerate myelin and to halt MS disease progression. In MS multiple factors are thought to be involved in remyelination failure, starting with a deficiency in OPCs because of impaired recruitment, or incomplete differentiation and maturation in the MS lesions. Several lines of evidence indicate a key role of microglia in these processes. Microglia regenerative expression profile involves genes related to phagocytosis, breakdown of myelin debris, as well as secretion of regenerative factors and tissue remodeling [36, 49] that can drive OPC differentiation [29, 32]. Here we show that TREM2 was highly expressed within active MS lesions by lipid-laden microglia/macrophages known to display an alternatively activated M2 profile and to promote the resolution of inflammation and clearance of myelin debris [5]. AL002a, by targeting and activating mouse TREM2, seems to enhance some of these pathways. It is not fully clear if enhanced remyelination in AL002a-treated *Trem2*^*+/−*^ mice is due to accelerated removal of myelin debris or if there is an additional TREM2-mediated signal from the microglia to recruit OPCs, thus giving an advantage in remyelination. Successive studies will further clarify potential microglia polarization or remodeling induced by AL002a. On the other end, it is well known that *Trem2*^*+/+*^ mouse microglia are very efficient in supporting complete remyelination in the acute CPZ model. This is different from what observed in MS, where a defect in TREM2 pathways has not been demonstrated, but still remyelination fails in most cases. A possible hypothesis could be that, in MS, microglia are chronically challenged by recurring bouts of demyelination and remyelination, suggesting that they might become exhausted and less able to respond over time. In this situation, microglia could be more responsive to an increase in TREM2 signaling via antibody-mediated activation. In future studies we could examine this further by either chronically feeding CPZ or looking in aged mice in which microglia functions could be defective.

Importantly, our study has also shown that AL002a treatment after CPZ is associated with reduced Nf-L plasma levels (a markers of axonal/neuronal damage), suggesting that increased remyelination also results in preserved axonal integrity, further proven by increased SMI31 staining in the CC of AL002a-treated mice. Altogether our study indicates that strategies aimed at targeting TREM2 on microglia in the CNS are feasible and might be a promising intervention in MS to promote microglia functions in clearing myelin debris, favoring the recruitment of OPCs, and their subsequent differentiation into mature myelin-generating oligodendrocytes, eventually leading to remyelination and axonal protection.

## Electronic supplementary material

Below is the link to the electronic supplementary material.

Supplementary material 1 (PDF 4613 kb)

Supplementary material 2 (XLSX 118 kb)

Supplementary material 3 (XLSX 10 kb)

Supplementary material 4 (XLSX 13 kb)

Supplementary material 5 (XLSX 514 kb)

Supplementary material 6 (XLSX 20 kb)
