# Supplementary material for: TREM2 activation on microglia promotes myelin debris clearance and remyelination in a model of multiple sclerosis
Source: Acta Neuropathol. 2020 Aug 9;140(4):513–34. doi: 10.1007/s00401-020-02193-z (PMC7498497; doi:10.1007/s00401-020-02193-z)
Supplement: Supplementary file 1 — Supplementary material 1 (PDF 4613 kb) [file 401_2020_2193_MOESM1_ESM.pdf]

SUPPLEMENTARY FIGURES

Supplementary Figure 1

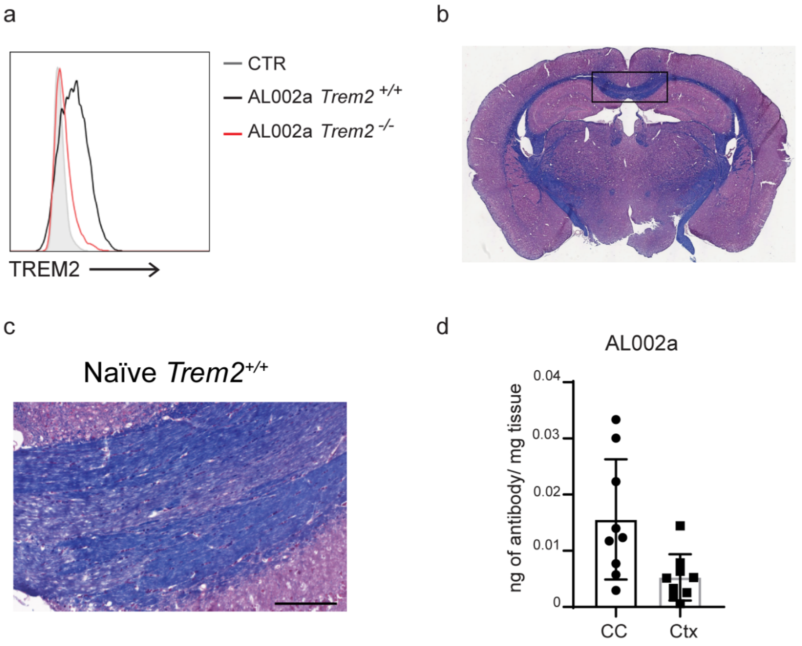

Supplementary Fig. 1. Anti-TREM2 antibody (AL002a) screening and CNS localization. (a) Flow cytometry analysis showing TREM2 mean fluorescence intensity (MFI) in *Trem2*$^{+/+}$ (black) and *Trem2*$^{-/-}$ (red) thioglycollate-induced macrophages (THG-Mac) treated with AL002a or isotype control (grey). At least 3 independent experiments were performed. **(b)** Solochrome cyanine staining of a representative coronal section of mouse brain. Box indicates the corpus callosum (CC), which is the main region of interest of this study. **(c)** Solochrome cyanine stained representative section of the CC of *Trem2*$^{+/-}$ naïve mice. Intact myelin is colored in dark blue. Scale bar= 300μm. **(d)** Quantification by Meso Scale Discovery (MSD) of AL002a antibody detected in the CC and cortex (Ctx) of *Trem2*$^{+/-}$ mice 48 hours after intraperitoneal injection of the antibody. AL002a N= 9.

Supplementary Figure 2

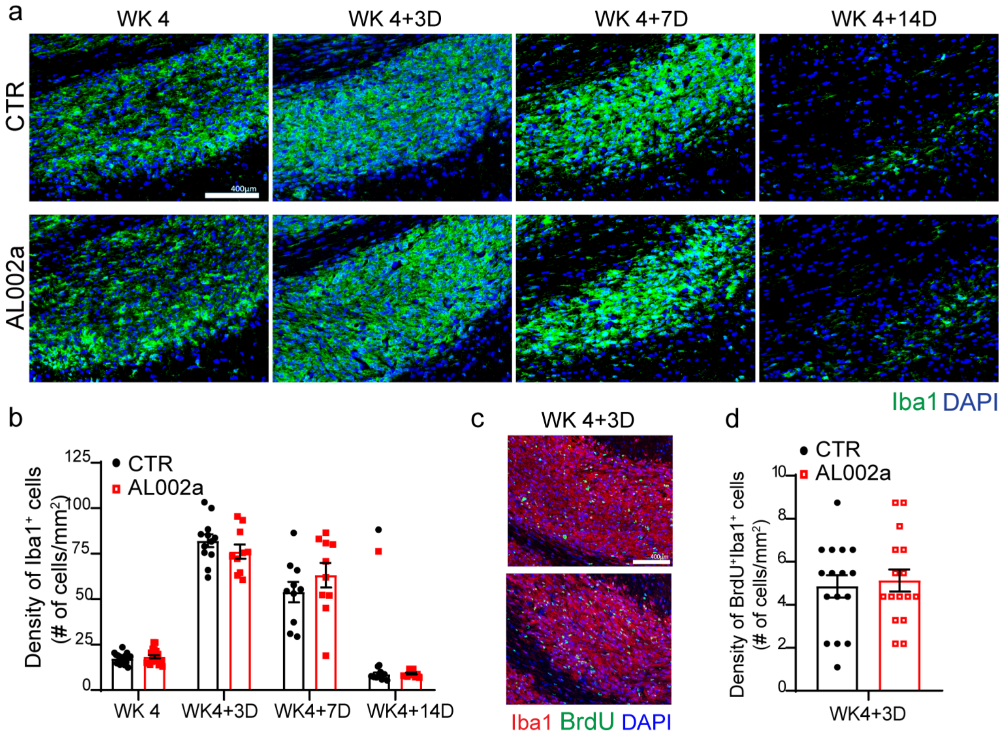

Supplementary Fig. 2. AL002a treatment does not affect microglia density and proliferation in CPZ-induced CNS demyelination in *Trem2*$^{+/-}$ mice. (a) Representative images of the CC of *Trem2*$^{+/-}$ mice treated with AL002a or CTR at week 4 (WK 4), week 4 plus 3 days (WK 4+3D), WK 4+7D and WK4+14D. Iba1 (green), DAPI (blue). Original magnification, 20X. Scale bar, 400µm. **(b)** Quantification of Iba1^{+} cells density in the CC. CTR group: WK 4 N= 5 mice, n= 20 fields; WK 4 +3D N= 6, n=12; WK 4 +7D N= 5, n=10; WK 4 +14D N= 5, n=10; AL002a treated group: WK 4 N= 5, n= 20; WK 4 +3D N= 5, n=10; WK 4 +7D N= 5, n=10; WK 4 +14D N= 6, n=12. **(c)** Representative images and **(d)** quantification of Iba1^{+} (red)/ BrdU^{+} (green) cells in the CC at 4 WK+3D. CTR group: N= 6, n=16; AL002a treated group: N= 5, n=16. Original magnification, 20X. Scale bar, 400µm.

Supplementary Figure 3

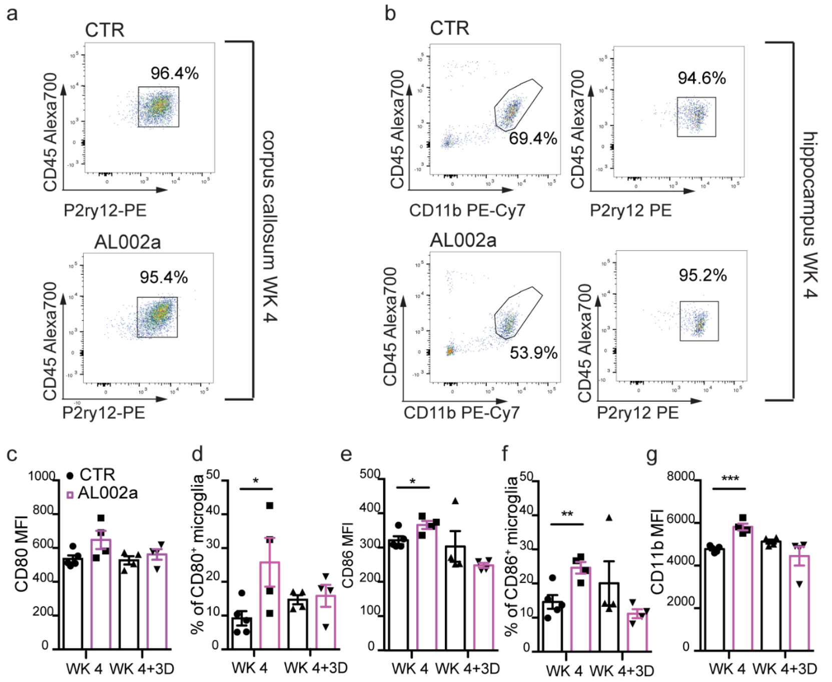

Supplementary Fig. 3. AL002a treatment induces activation of microglia in the hippocampus after CPZ. (a) FACS analysis of microglia isolated at week 4 (WK 4) from the CC of *Trem2*$^{+/-}$ mice treated with AL002a or CTR antibodies. CD11b^{+}CD45^{int} cells were also identified as P2yr12^{+} cells upon doublet discrimination and dead cell exclusion. **(b)** Representative dot plots of microglia isolated from the hippocampus (HP) at WK 4 and identified as CD11b^{+}CD45^{int} and P2yr12^{+}cells. **(c, e, and g)** Quantification of surface CD80, CD86, and CD11b MFIs at WK 4 and WK 4 +3D in microglia isolated from the HP. **(d and f)** Analysis of the percentage of cells expressing CD80 and CD86. WK 4 CTR group N = 4 and AL002a treated group N= 5; WK 4+3D *$P < 0.5$, **$P < 0.01$, ***$P < 0.001$ two-tailed unpaired Student's t test.

Supplementary Figure 4

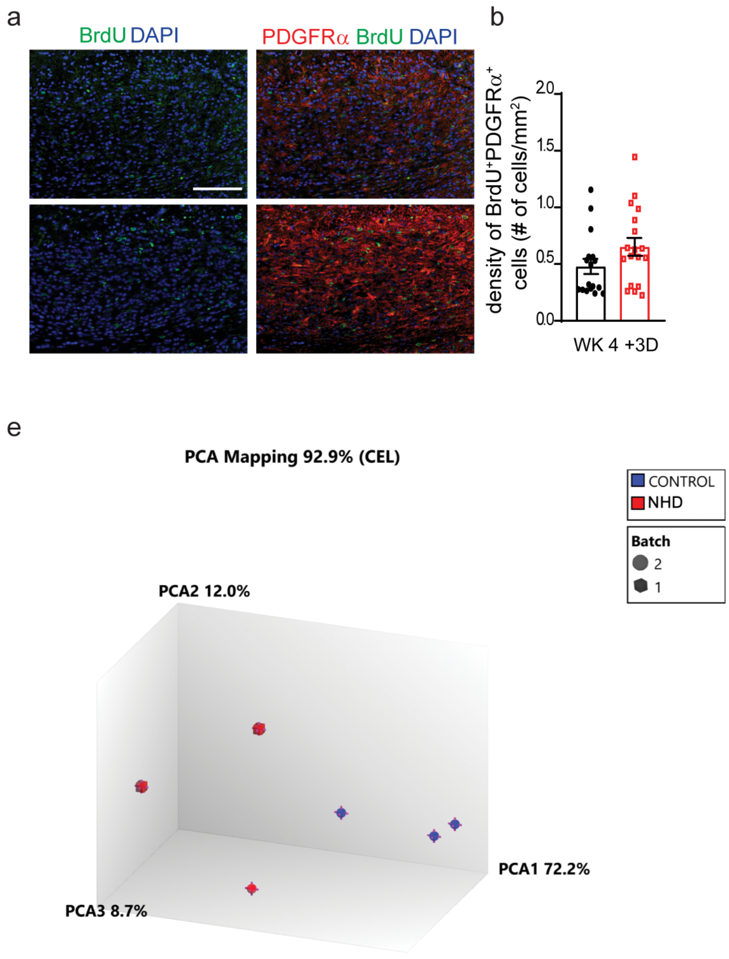

Supplementary Fig. 4. AL002a does not impact on PDGFRα^{+} OPC proliferation.
PCA analysis of Nasu-Hakola disease (NHD) and healthy control macrophages. (a) Representative images of PDGFRα^{+} (red), BrdU (green) and DAPI (blue) in the CC at WK 4 +3D and WK 4+7D. Original magnification, 20X. Scale bar, 400µm. **(b)** Quantification of the density of PDGFRα^{+} BrdU^{+} cells. CTR group: WK 4+3D N= 6, n=24; WK 4+7D N= 5, n=20; AL002a treated group: WK 4+3D N= 6, n= 24; WK 4 +7D N= 5, n=20. **(c and d)** qRT-PCR quantification of mRNA levels of **(c)** oligodendrocyte transcription factor (*Olig*2) and **(d)** 2',3'-Cyclic nucleotide 3'-phosphodiesterase (*Cnp*) in the corpus callosum (CC) and the hippocampus (HP) of *Trem2*$^{+/-}$ mice treated with AL002a antibody or CTR. Tissues were collected at WK 4+7D. CTR group: N= 5, AL002a treated group: N= 5. *$P < 0.5$, two-tailed unpaired Student's t test. **(e)** Principal component analysis of NHD subjects (in red) and healthy control subjects (in blue).
